# Evaluation of five microbial and four mitochondrial DNA markers for tracking human and pig fecal pollution in freshwater

**DOI:** 10.1038/srep35311

**Published:** 2016-10-13

**Authors:** Xiwei He, Peng Liu, Guolu Zheng, Huimei Chen, Wei Shi, Yibin Cui, Hongqiang Ren, Xu-Xiang Zhang

**Affiliations:** 1 State Key Laboratory of Pollution Control and Resource Reuse, School of the Environment, Nanjing University, China; 2 College of Agriculture, Environmental and Human Sciences, Lincoln University, Missouri, USA; 3 Jiangsu Key Laboratory of Molecular Medicine, School of Medicine, Nanjing University, China

## Abstract

This study systematically evaluated five microbial and four mitochondrial DNA (mtDNA) markers, including sensitivities and specificities under PCR method, and fecal concentrations and decay rates in water under qPCR method. The microbial DNA markers were the three human-associated (BacH, HF183 and B.adolescentis) and two pig-associated (Pig-2-Bac and L.amylovorus), while the mtDNA ones were two human- (H-ND6 and H-ND5) and two pig-associated (P-CytB and P-ND5). All the mtDNA markers showed higher sensitivity (100%) than the microbial ones (84.0–88.8%) except Pig-2-Bac (100%). Specificities of the human mtDNA markers (99.1 and 98.1%) were higher than those of the human-associated microbial ones (57.0–88.8%). But this pattern was not observed in the pig-associated markers where Pig-2-Bac had 100% specificity. The reliability of H-ND6 and H-ND5 was further evidenced to identify locations of the most polluted within the Taihu Lake watershed of China. In general, the microbial DNA markers demonstrated a higher fecal concentration than the mtDNA ones; increasing temperature and sunlight exposure accelerated significantly the decay of all the DNA markers. Results of this study suggest that DNA markers H-ND6, H-ND5, and Pig-2-Bac may be among the best for fecal source tracking in water.

Fecal contamination in water often leads to serious public health issues, and fecal source tracking (FST) methods are a promising tool to identify the host sources of fecal pollution for taking effective steps to mitigate or eliminate the pollution sources^1^,^2^,^3^,^4^. Microbial reference library-dependent method used to be developed for FST, but it is labor intensive and expensive for requiring a large number of isolates to generate reliable results^1^. Chemical markers were also used for FST and some detergent compounds have been identified, including whitening agents, sodium tripolyphosphate and long chain alkylbenzenes as indicator markers. But these tracers are only human-origin indicators and can’t be used to differentiate the origins of animal feces^3^. Recently, FST methods targeting host-associated genetic markers have gained great attention, as the procedures do not require construction of libraries from known host sources of a potential pollution and are convenient for trace analysis by polymerase chain reaction (PCR) assays^5^. Based on their origins, FST genetic markers can be classified as microbial DNA and mitochondrial DNA (mtDNA) ones. The former are mainly 16S rRNA gene fragments of gut microbes specific to or highly associated with a host species, while the latter are host-specific mtDNA sequences of host cells.

Over the past two decades, many microbial DNA and mtDNA markers have worldly been developed for FST^4^,^6^, since the pioneer work of Field and co-worker^7^,^8^. The majority of microbial DNA markers has been derived from anaerobic bacteria of order *Bacteroidales*, as recently reviewed by Roslev and Bukh^5^. Lately, other dominant gut microorganisms have also used in FST, including bacteria of genera *Bifidobacterium*^9^,^10^,^11^,^12^ and *Lactobacillus*^13^. Moreover, with the availability of mtDNA genomes of human and several livestock animals that are common sources of fecal pollution, mtDNA-based markers have emerged to be effective in differentiating the sources of fecal pollution^14^,^15^,^16^. Compared with mtDNA markers, it is generally believed that microbial DNA markers are more abundant in host feces. But mtDNA markers are geographically stable, because host mtDNA is not as easily influenced by animal diets, but environmental microorganisms as gut microbiota would be^5^. However, there is little study to systematically compare these two types of FST markers.

It is not unusual that the performance of a FST marker varies from location to location, demonstrating a geographical instability^17^. Most of FST markers were developed in the West, including America and France. It remains unknown the application of these markers in water environments of China. On the other hand, rapid development of livestock farming and population growth has led to serious fecal pollution in China. The Taihu Lake watershed is suffering from eutrophication, and fecal pollution is the great contributor. There is an urgent need to develop or identify effective FST markers to prioritize impaired waters of China for remediation. An ideal FST marker is to have a relatively high content in polluting matrix base on the guideline document of the United States Environmental Protection Agency (2005), to demonstrate a consistent decay rate in different types of waters and habitats, and to be geographically stable. Although the current microbial DNA and mtDNA markers are reportedly abundant in host feces, their decay in water environments is still not well understood^5^,^18^ and knowledge of their geographical stability is limited.

It is evidenced that the decay rate of a DNA marker is affected significantly by water temperature, sunlight exposure, and origin of the DNA marker^19^,^20^,^21^. Several previous researches on the persistence of DNA markers using microcosms reported that both microbial DNA and mtDNA markers could persist longer at lower temperature and without sunlight exposure^21^,^22^,^23^ and that the lifespan of DNA markers could range from days to weeks in water, depending on the environmental conditions^21^,^22^,^23^. However, it has been lacking to symmetrically compare decay rates of different DNA markers (microbial vs. microbial, microbial vs. mtDNA, and mtDNA vs. mtDNA) under various conditions for making an informed selection of an effective FST marker(s).

Therefore, the aim of this study was to systematically evaluate five microbial DNA markers (BacH, HF183, B.adolescentis for human and Pig-2-Bac, L.amylovorus for pig) and three mtDNA markers (H-ND5 for human andP-CytoB, P-ND5 for pig) that were selected as the benchmark FST markers from the literature. In parallel, a human mtDNA marker, H-ND6, developed in this study was included for the evaluations. All the markers’ sensitivities, specificities, and fecal concentrations were determined using the fecal and water samples collected from the Taihu Lake watershed of China; their decay rates under different environmental conditions were examined in the laboratory and field microcosms; and finally, the human-associated microbial DNA and mtDNA markers were further evaluated in identifying locations of the most polluted along Taige River of the Taihu Lake watershed. Knowledge gained from this study was expected to enhance water quality management of the Taihu Lake watershed in particular and FST in general.

## Results

### Sensitivities and Specificities of the DNA markers

Among the 5 human-associated markers, mtDNA markers (H-ND6 and H-ND5) had a sensitivity of 100% and specificities of ≥98.1%, while the microbial DNA ones (BacH, HF183 and B.adolescentis) displayed sensitivities of ≤92% and specificities of ≤88.8% (Table 1). However, the distinction between mtDNA and microbial DNA markers was not seen for the 4 pig-associated markers. As shown in Table 1, microbial DNA marker Pig-2-Bac had 100% sensitivity and specificity, being superior to the other three pig-associated; although both mtDNA markers P-ND5 and P-CytB had 100% sensitivity, their specificities were only 84.1% and 68.2%, respectively. The performance of L.amylovorus was intermediate.

### Concentrations of the DNA markers in feces

**Figure 1:**
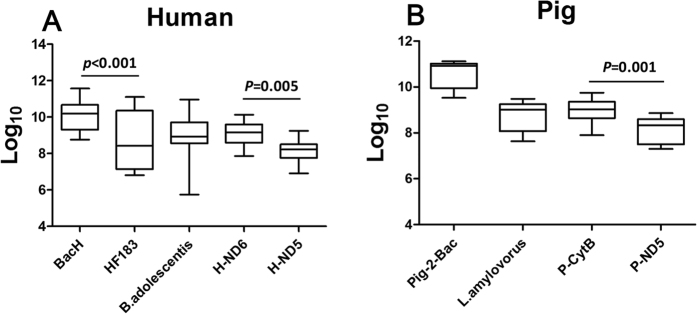
Boxplot representation of the concentrations of the DNA markers in 1 g of individual feces samples. (**A**) The human-associated markers in human feces; (**B**) The pig-associated markers in pig feces. The median (line within the box) and quartiles are represented. Whisker caps represent the maximum and minimum concentrations of the DNA markers. The F statistics and degree of freedom (df) for the ANOVA test were 9.275 and 4, respectively.

The concentrations (log_10_ copies/g feces) of all the 9 markers in individual fecal samples were estimated by the corresponding qPCR assays. The results showed that the concentrations in feces seemed to have a decreasing trend in the order: BacH (10.08 ± 0.82 log_10_ copies/g), H-ND6 (9.08 ± 0.58 log_10_ copies/g), B.adolescentis (8.97 ± 1.13 log_10_ copies/g), HF183 (8.80 ± 1.56 log_10_ copies/g), and H-ND5 (8.13 ± 0.57 log_10_ copies/g), for the 5 human-associated markers (Fig. 1A and Table S1), and Pig-2-Bac (10.56 ± 0.58 log_10_ copies/g), P-CytB (8.96 ± 0.54 log_10_ copies/g), L.amylovorus (8.72 ± 0.63 log_10_ copies/g), and P-ND5 (7.61 ± 0.57 log_10_ copies/g), for the 4 pig-associated ones (Fig. 1B and Table S1). Notably, the markers with the highest concentration for human (BacH) and for pig (Pig-2-Bac) were both *Bacteroidales*-origin^24^,^25^ and the human mtDNA markers demonstrated lesser variations among individual fecal samples than their microbial counterparts (Fig. 1A,B).

Interestingly, the DNA marker concentrations were negatively correlated with their sizes, i.e. the length of PCR amplicons, when the markers were generated from the same genome. This negative correlation was observed for mtDNA markers H-ND6 (9.08 ± 0.58 log_10_ copies/g, 97 bp) vs. H-ND5 (8.13 ± 0.57 log_10_ copies/g, 195 bp), and P-CytB (8.96 ± 0.54 log_10_ copies/g, 116 bp) vs. P-ND5 (7.61 ± 0.57 log_10_ copies/g, 196 bp), as well as for *Bacteroidales*-origin markers BacH (10.08 ± 0.82 log_10_ copies/g, 93 bp) vs. HF183 (8.80 ± 1.56 log_10_ copies/g, 541 bp) (Fig. 1A,B).

### Concentrations of the human-associated DNA markers in sewage wastewater

As expected, all sewage water treatment plant (SWTP) samples collected from the influent and effluent flows were positive for all the five human-associated markers. The concentrations (log_10_ copies/liter) of the markers were measured by the qPCR assays (Table 2). For each marker, no significant difference (F = 0.179, df = 1, *p* = 0.674) in concentration was observed in the samples from the summer vs. those from the winter; however, there was a significant difference (F = 79.877, df = 1, *p *< 0.001) in the samples from the influent vs. those from the effluent. In summer, the concentrations of the markers in the influent were generally 100–6000 times higher than those in the effluent, but this difference reduced to 50–100 times in winter (Table 2). Among the five markers, BacH and B.adolescentis showed the highest mean concentrations in the influent at 8.79 and 8.67 log_10_ copies/liter, respectively, followed by HF183 and H-ND6 with mean concentrations at 8.18 and 7.58 log_10_ copies/liter, respectively. H-ND5 showed the lowest mean concentration at 6.10 log_10_ copies/liter.

### Decay of the DNA markers in water

**Figure 2:**
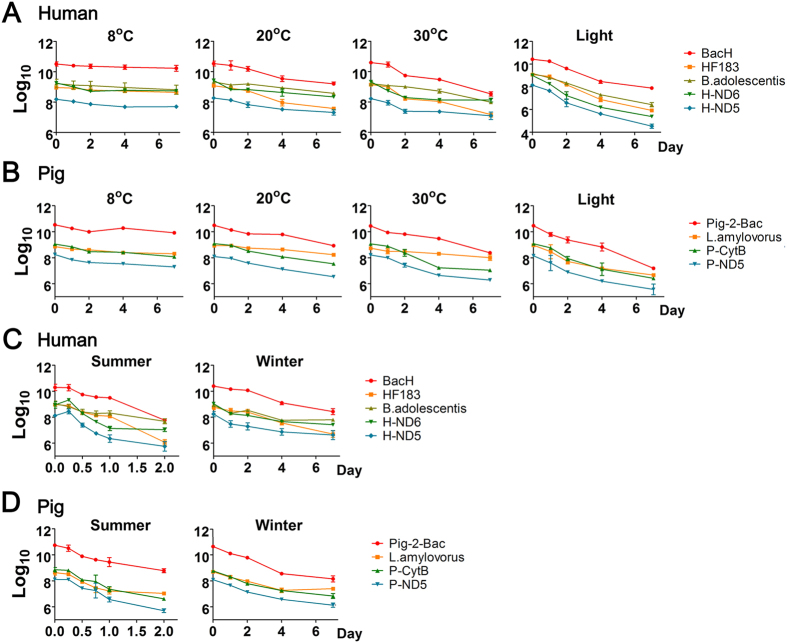
Decay curves of the DNA markers under different conditions. (**A**) The human-associated markers in the laboratory microcosm; (**B**) The pig-associated markers in the laboratory microcosm; (**C**) The human-associated markers in the field microcosm; (**D**) The pig-associated markers in the field microcosm. The data are shown as log_10_ copies/L water.

Overall, the copy numbers of all the 9 markers decreased over time in the microcosms (Fig. 2). As shown in Table S2, the slowest decay rate of each marker was seen at 8 °C in the dark. When the temperature rose to 20 °C or 30 °C, decay rates of all DNA markers increased significantly (all *p *< 0.05). We further calculated the T_90_ values (time needed for 90% reduction of the initial concentration) to show how long DNA marker could persist in water environments (Table 3). Among the human-associated markers, the T_90_ values of *Bacteroidales* markers BacH and HF183 were 22.71 d and 21.12 d, respectively at 8 °C, they decreased to 4.95 d and 4.44 d at 20 °C and o 3.38 d and 3.47 d at 30 °C, respectively. In comparison, the mtDNA markers H-ND6 and H-ND5 and *Bifidobacterium* marker B.adolescentis persisted shorter at 8 °C but longer at 20 or 30 °C. On the other hand, among the pig-associated markers, *Lactobacillus* marker L.amy appeared to be less sensitive to the higher temperature than mtDNA markers P-CytB and P-ND5 or *Baceroidales* marker Pig-2-Bac (Table 3). In the laboratory microcosms, all the 9 markers reached their fastest decay rates at 20 °C in the light, where all the markers had a similar T_90_ at ~2 to ~3 d, which was a significant reduction (ranging from ~2 to ~7 d decrease) from their corresponding T_90_ at 20 °C without the light (Table 3).

The field microcosm illustrated that the T_90_ values of all 9 markers decayed significantly faster (*p *< 0.001) in the summer than in the winter. But, markers B.adolescentis and L.amylovorus had higher T_90_ values than any other human- and pig-associated markers, respectively in both seasons (Table 3). It was in agreement with the aforementioned observations of these two markers in the study of laboratory microcosms.

Interestingly, when derived from the same origin, DNA markers (e.g. BacH and HF183, H-ND6 and H-ND5, or P-CytB and P-ND5) tended to have no significantly different decay rates under various conditions; while markers of different origins (e.g. BacH and B.adolescentis, H-ND6 and B.adolescentis, or P-CytB and L.amylovorus) tended to decay at significantly different rates under these conditions (Tables S3–S8).

### Detection of the human-associated markers in Taige River

**Figure 3:**
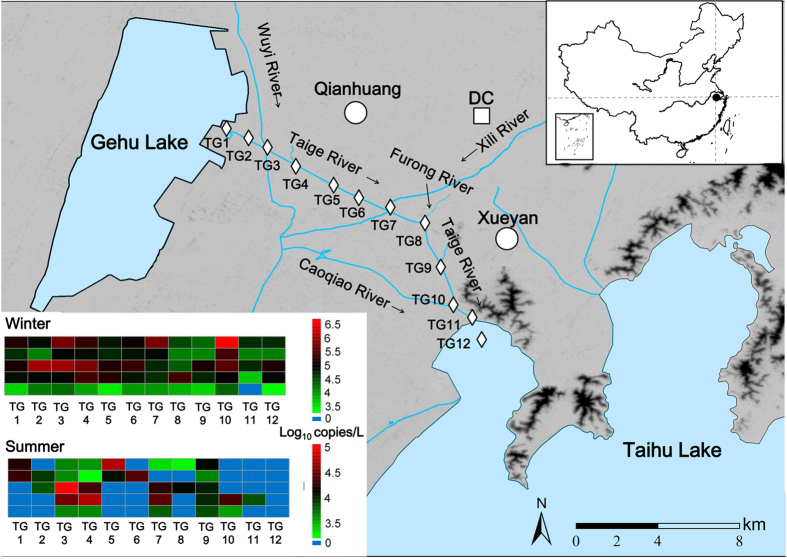
Map of the sampling sites along Taige River with a heatmap of the concentrations of the human-associated DNA markers at each sampling site. The map was created using ArcGIS 10.0 (http://www.esrichina-bj.cn/softwareproduct/ArcGIS/). The rhombus refers to sampling site, the square refers to sewage water treatment plant, and the circle refers to town.

Twelve sampling sites were selected and the five human-associated markers were measured along Taige River of the Taihu watershed (Fig. 3). In the winter, the human-associated markers were almost detected at sampling sites except that H-ND5 was absent at TG11. But in summer, the detection rates of the DNA markers were lower (41.7–58.3%), and the presences of the 3 human-associated microbial DNA markers in the water samples were not similar with each other; by contrast, the pattern of the presence and absence of mtDNA markers H-ND6 and H-ND5 were identical for all the sampling sites except TG11, from where the water samples were, like in the winter, negative for H-ND5 but positive for H-ND6.

Concentrations of the DNA markers varied distinctly among the sampling sites. At sites TG3, 4, 7, and 9, more than four markers were detected simultaneously at each site in both winter and summer at levels up to 5.95 log_10_ copies/L. At sampling site TG10, two mtDNA markers were detected in the summer, but all five markers were detected at relatively high concentrations (up to 6.65 log_10_ copies/L) in the winter. At other sites, e.g. TG1, 6, and 8, only one or two microbial markers were detected in the summer, and the markers detected in the winter were at relatively lower concentrations (Fig. 3 and Table S9).

## Discussion

This study firstly and systemically evaluated the selected benchmark microbial DNA and mtDNA markers, based on their sensitivities, specificities, fecal concentrations, and decay in water environments. New information gained through this study may be useful for tracking human and pig fecal pollution in the Taihu Lake watershed of China in particular and for FST in general.

Knowledge regarding the geographical stability of a DNA marker, mainly its sensitivity and specificity, is critical for widespread application of the marker in FST^17^,^26^. In our study, only human mtDNA marker H-ND5 and pig-associated microbial DNA marker Pig-2-Bac appeared to be geographically stable, as demonstrated by that their performances in this study were in agreement with those seen in the previous studies conducted in the West. The sensitivity and specificity of H-ND5 in this study were 100 and 98.1%, respectively, which were comparable with those (100% sensitivity and 100% specificity) observed in USA^27^. On the other hand, Pig-2-Bac developed in France showed 100% sensitivity and specificity, being the same as seen in France^28^. Furthermore, the 100% sensitivity and 99.1% specificity of mtDNA marker H-ND6 developed in this study appeared to be as good as that of H-ND5 (Table 1). However, geographical stability of H-ND6 was yet to be examined using fecal samples from broaden locations.

Overall, the performances of human-associated microbial DNA markers BacH, HF183, and B.adolescentis were inferior to those of human mtDNA markers H-ND6 and H-ND5 (Table 1). Although the three microbial markers exhibited a sensitivity greater than 84%, which is comparable with those seen in USA^4^, France^13^,^28^, and Australia^24^, the markers demonstrated a somewhat lower specificity (≤88.8%) in this study, compared with those (≥94.5%) observed in other countries^6^,^11^,^24^,^29^. The consistent sensitivities of BacH, HF183, and B.adolescentis in different regions indicate that these markers may globally distributed in the target host species (i.e. human or pig). However, it is difficult to simply compare the data from different laboratories with various complex conditions. The relatively low specificities of the three markers shown in this study just suggested a possible geographical instability of marker specificity. The existence of these microbial DNA markers in the feces of several non-target animal species may lower their application value for fecal source tracking in China.

In contrast to human mtDNA markers, pig mtDNA markers P-CytB and P-ND5 demonstrated a lower specificity (68.2% and 84.2%, respectively) than that of microbial DNA marker Pig-2-Bac (100%), but comparable with that of another microbial maker L.amylovorus, although the sensitivities of both P-CytB and P-ND5 were 100% (Table 1). It is not surprising that the two pig mtDNA markers could be found in some human fecal samples, because pork was the major meat in the diets of people around Taihu Lake region and could result in detectable pig mtDNA (subsequently the mtDNA markers) in human waste. It is however somewhat surprising that P-CytB could be found in fecal samples from almost all the animal species tested except for those of chicken and that, in fewer instances, P-ND5 in feces of some non-target animal species (Table 1). The relatively low specificities (≤ 84.1%) of P-CytB and P-ND5 can be at least explained in part by that their PCR target sequences are relatively conserved among mtDNAs of different host species. Based on the sequence comparisons of the mtDNA regions targeted by the PCR primers of P-CytB or P-ND5, there is a high degree of similarity, ranging from 71.4% to 93.8% (Fig. S1) between the mtDNA sequence of pig (target species) and those of human and other animals (non-target species). The sequence similarity is particularly high between P-CytB primers and the corresponding mtDNA sequences of dog (93.8%), cattle (87.5%) and goat (85.4%), which were largely correlated with the positive rates of P-CytB in the feces of these three animal species (Table 1). Thus, pig-associated mtDNA marker may need to be redesigned for reducing the cross reaction of its PCR primers with non-pig mtDNA genomes, which can be achieved by targeting a more variable region of the pig mtDNA. In conclusion, the 100% sensitivity and specificity of Pig-2-Bac made it a more reliable marker than any other pig-associated makers tested.

The concentration of a DNA marker in target feces and its decay in water are another two crucial factors affecting successful detection of the marker in feces-polluted water. Apparently, for the same pollution level, the concentration of a marker in feces-polluted water is positively correlated with the marker’s abundance in the host intestines (subsequently the feces) and negatively with its decay rate in water. Bacteria of order *Bacteroidales* and genera *Bifidobacterium* and *Lactobacillus* were reportedly among the dominant flora in guts of humans and pigs^12^. Our data showed that *Bacteroidales* might be the most dominant host-specific microbes, as both the most abundant human-associated BacH and pig-associated Pig-2-Bac were *Bacteroidales*-origin. However, it was shown that the fecal concentrations of the microbial DNA markers of *Bacteroidales, Bifidobacterium* or *Lactobacillus* varied greatly among individuals and some target individuals were even undetectable for the corresponding markers, except Pig-2-Bac (Fig. 1 and Table 1). By contrast, all mtDNA markers demonstrated relatively small variations in fecal concentrations and were 100% positive in the target individuals, although their concentrations were lower than the microbial DNA markers (Fig. 1).

Our data also demonstrated that a marker’s fecal concentration measured by a qPCR assay was negatively correlated with the size of the marker, given that the same genome was targeted. This finding was illustrated by the results of qPCR assays for BacH vs. HF183, H-ND6 vs. H-ND5, and P-CytB vs. P-ND5. The underlying assumption has been that amplifying longer DNA fragments, the markers in this case, increases the probability with which the DNA polymerase encounters a damage that interferes with the elongation during the PCR amplification^30^. However, the lengths of the qPCR products seemed not to obviously influence the decay rates of the DNA markers, as seen for the aforementioned four pairs of markers under both the laboratory and field conditions (Tables S3–S8). Therefore, it is recommended to select or to develop short DNA markers for qPCR assays in FST to reduce potential false-negative detection of feces-polluted water due to low copy number of the markers for the PCR amplifications.

The concentrations of the human-associated markers in SWTP wastewater samples (Table 2) demonstrated again that a marker’s concentration in water was negatively correlated with the size of the marker and that the concentrations of mtDMA markers were generally lower than those of the human-associated microbial DNA markers, regardless of the source (influent vs. effluent) and season (winter vs. summer). In other words, BacH, HF183, and B.adolescentis were more likely to be detected than H-ND6 and H-ND5 in sewage-polluted water. However, the relatively high concentrations of the microbial DNA markers might due to their relatively low specificities (i.e. cross reactions with fecal DNA of non-target feces).

The laboratory microcosm was used to determine the effects of temperature and light exposure (mimic of sunlight) on the decay of all the 9 markers in water. It was found that increasing temperature significantly accelerated the decay rates of both microbial DNA and mtDNA markers in water environments (Fig. 2; Tables 3 and S2). This result was in accordance with those of previous studies^20^,^21^,^23^. Light exposure was found in this study to significantly increase the decay of all the markers and the impact was so profound that it could almost overwrite the differences among the markers observed in dark at 8, 20, or 30 °C (Table 3). The accelerating effects of temperature and sunlight on the decay of the DNA markers were further evidenced in our field microcosm, where all the DNA markers decayed faster in the summer (higher temperature and sunlight exposure in combination) than in the winter or in the laboratory settings. Several previous studies reported that different DNA markers decayed differently in response of sunlight exposure^19^,^22^,^31^,^32^. This is supported by our data of the field microcosm, where mtDNA and *Baceroidales* DNA markers of both the human- and pig-associated decayed at a similar rate, but faster than B.adolescentis and L.amylovorus, regardless of the seasons. Since the *Bifidobacterium* and *Lactobacillus* markers appeared relatively stable in water, developing new FST markers derived from these two groups of microorganisms with higher specificity and sensitivity may be worth pursuing in future study.

With the heavy urbanization of the region of Taihu Lake watershed in recent years, human fecal pollution in the watershed has become a major concern of the public health and sustainable aquaculture. The Chinese central and local governments have allocated funds for control and remediation of the pollution. We used the five human-associated DNA markers to identify possible polluted locations along Taige River (an on-going project) in order to prioritize investments for remediation. In the winter, almost all the human-associated markers could be detected at all the sampling sites along Taige River (Fig. 3A and Table S7), indicating there might be human fecal contamination in this river. As expected, the presence and absence of H-ND6 and H-ND5 were almost identical for all the sampling sites except TG11, since the sensitivity and specificity of both the markers had been almost perfect. The inconsistent results of H-ND6 and H-ND5 for site TG11 may be explained by the fact that H-ND6 is a shorter marker and therefore, more abundant for the qPCR detection as discussed above. By contrast, although targeting the same genome, microbial DNA markers BacH and HF183 did not always present a comparable detection of human fecal pollution, which might be due to their cross reaction with feces of other animal species (Table 1). From the point of view of effective water quality management, it is critical to prioritize locations for remediation. Given the near-perfect sensitivity and specificity of H-ND6 and H-ND5 and the relatively short persistence of DNA markers in summer, the relatively high concentrations of the two markers found at sites TG3, TG4, TG7, TG9, and TG10 in the summer suggested that these sites might have relatively more fecal loads in need of taking management steps. This finding was supported by our field survey. As indicated in Fig. 3, sites TG3, TG7, and TG10 were the confluences of Taige River with Wuyi River, Xili River and Caoqiao River, respectively. The three rivers might have brought extra human fecal loads into the Taige River as they all run through populated towns. Sites TG4 and TG9 were at the entrance of two small branches of Taige River stretching from rural riverside communities where municipal sewage treatment infrastructure was not available. Local residents might also discharge their wastewater into domestic open-air septic tanks, and some faulty ones could easily cause wastewater overflow. It is worth mentioning that the human mtDNA markers in the river water were unlikely from sloughed skin or hair from swimming activities since local people seldomly swim in this river due to the poor water quality.

The presence of BacH, HF183, and B.adolescentis at sites TG1, TG2, TG5, TG6, and TG8 might resulted from cross reactions between the their PCR primers with fecal DNA from feces of poultry or other animals, as seen in the laboratory tests (Table 1). This assumption was supported by the information of our field survey, during which free-ranged poultry were spotted on the river banks and additionally, local farmers had the tradition of using animal manure as fertilizer for their crop fields, which might serve as a non-point source of fecal pollution to Taige River.

## Conclusion

In summary, this study found that mtDNA markers appeared to have less variation among individuals, and DNA markers of Baceroidales-origin may be the most abundant for PCR detection. Human mtDNA markers H-ND6 and H-ND5 may be more reliable and superior to the human-associated microbial DNA markers BacH, HF183, and B.adolescentis. The human mtDNA marker H-ND6 developed in this study presented a relatively low concentration of human fecal pollution where H-ND5 failed to be identified; however, complete value of H-ND6 needs to be further verified using fecal samples from broaden locations. The microbial DNA marker Pig-2-Bac may be a reliable pig-associated FST marker, while pig mtDNA markers P-CytB and P-ND5 were not specific enough to differentiate pig mtDNA from at least those of cattle, dog, and goat. Therefore, we propose to develop a new pig mtDNA marker by targeting a more variable region of pig mtDNA. DNA markers of *Bifidobacterium*- and *Lactobacillus*-origin may be more stable in water in response of increasing temperature and sunlight exposure, compared with the markers of mtDNA- and *Baceroidales*-origin. A DNA marker’s concentration in water measured by a PCR assay was negatively correlated with its size; therefore, shorter DNA markers may be used to enhance PCR detections of fecal pollution. In conclusion, markers H-ND6, H-ND5, and Pig-2-Bac may be among the best for fecal source tracking. Both microbial DNA and mtDNA markers have their own merits and the toolbox method using these two types of FST markers is recommended for accurate identification of the pollution sources in water.

## Methods

### The DNA markers

A total of nine DNA markers, including five human-associated and four pig-associated, were used in this study (Table 4), among which eight markers (four each for human and pig) were previously developed and successfully used in the West. The human-associated mtDNA marker H-ND6 was developed in this study. The corresponding PCR primers were designed by aligning human, porcine, bovine, ovine, dog, and chicken mtDNA (GeneBank accession no. NC_012920, NC_000845, AF492351, NC_001941, NC_002008, and NC_001323, respectively) using the Clustal X software (Conway Institute, Ireland). The human-specific primer hybridization sites were identified within the ubiquinone oxidoreductase core subunit 6 (NADH6) that has great variation with the other mtDNAs. Primers were then designed using the Clone Manager Professional 8 (Scientific & Educational Software, USA) before subjected to a specificity test with the BLASTN program (http://www.ncbi.nlm.nih.gov/).

### The collection of fecal and water samples

Fresh human fecal samples were collected from healthy individuals between ages 10 and 50 (n = 25) and fresh individual pig fecal samples from 5 different pig farms (n = 25). Additional fecal samples were obtained from seven other species (bovine, goat, dog, chicken, duck, goose, and kept on ice during transportation, and then stored at −80 °C within 4 hours of collection prior to DNA extraction. The experimental protocol was approved by Research Review Board of Nanjing University and the methods involving human subjects (fresh human fecal samples) were performed in accordance with the Declaration of Helsinki. All human fecal sample collection was approved by the local ethics committee of Nanjing University, and informed consent was obtained from each donor. Animal fecal samples were collected and treated in strict accordance with the National Institutes of Health Guide for the Care and Use of Laboratory Animals (China). This study did not involve any experiments on live vertebrates, or human or animal tissue.

Wastewater samples (n = 12) were collected from the influent and effluent of the sewage water treatment plant (SWTP) Dachang (three-Tank Oxidation Ditch, 100000 t/d) located at town Wujin, where sewage wastewater from towns Qianhuang and Xueyan was treated. Furthermore, water samples (n = 72) were collected from 11 sampling sites along Taige River and one site off the river in Taihu Lake (Fig. 3). All the locations were sampled three times each in December, 2013 and in July, 2014. The river extends over 20 km from Gehu Lake to Taihu Lake. It runs through the urbanized areas including towns Qianhuang and Xueyan, with populations of ~42,000 and 28,000, respectively. The Taige River subwatershed covers ~68 km^2^ of agricultural field for crops, mainly rice, wheat, and canola. Our previous research using nitrogen isotope showed that Taige River was contaminated by human feces (data not published). There was no recent census information available for livestock and poultry operations in this area; however, free-ranged chickens, ducks, and gooses as well as small-scale pig and goat feedlots were spotted during a field survey in this area.

All surface water and wastewater samples were taken using 10 L sterilized bottles and filtrated through 0.45-μm hydrophilic polyethersulfone membranes with a vacuum. The membranes with intercepted materials from water samples were stored at −80 °C before usage. The volume of filtrate (~200 mL SWTP influent, ~1.5 L SWTP effluent, and ~800 mL river water through one membrane) was recorded for each sample and used for calculating the concentrations of the DNA markers.

### Laboratory and field microcosms

The laboratory and field microcosms were designed to evaluate the decay of the DNA markers under the controlled and natural conditions, respectively. For the laboratory study, fresh human or pig feces were diluted to 1 g/L with the surface water obtained from Taige River and the triangular flasks containing 1 L of the fecal suspension were incubated aerobically under 1) 8 °C, in dark; 2) 20 °C, in dark; 3) 30 °C, in dark; or 4) 20 °C with artificial light (the intensities of the UVA, UVC, LUX, and PAR of the light were 0.2 mW/cm^2^, 10.2 μW/cm^2^, 9680 lux, and 129 μmol/m^2^/s, respectively, 12 h on/12 h off).

The field microcosm study was performed with dialysis tube stripe (MW: 3,500 D, MD44, Biosharp, USA). The tube stripes were separately sealed, containing human or pig fecal suspensions (1 g/L), and incubated 30 cm below the water surface of Furong River of the Taihu Lake watershed (Fig. 3). The experiments were conducted in the early September and late November of 2014, and the parameters of the weather and river water conditions were recorded (Table S10). The fecal suspension (20 ml each) was collected in triplicate from each group on day 0, 1, 2, 4, 7 and centrifuged at 12,000 rpm for 20 min. The pellets were stored at −80 °C until DNA extraction.

### DNA extraction and PCR assays

Total DNA was extracted from individual fecal sample, pellets of centrifugated suspension, and clogged membranes using QIAamp DNA Stool Mini Kit (QIAGEN, Hilden, Germany) according to the manufacturer’s instructions. The concentration and purity of the DNA extracted from each sample were determined microspectrophotometrically (NanoDrop^®^ ND-2000, NanoDrop Technologies, Wilmington, DE, USA).

Conventional-PCR assays were conducted to measure the sensitivities and specificities of the DNA markers according to Ahmed *et al*.^6^. The PCR assays were performed in a Veriti 96-Well Thermal Cycler (Applied Biosystems, Life Technologies). Amplifications were performed in duplicate in 20 μl reaction mixture containing 10 μl of Easytaq SuperMix (TransGen Biotech, China), 4 μl of template DNA, 0.5 μl of each primer (250 nM) and 5 μl of ddH_2_O. The PCR primers for each marker were listed in Table 4. The corresponding PCR thermocycle for each marker was detailed in Table S11. Each sample was tested twice and considered positive only when both the PCR amplifications generated the expected size of amplicon as visualized by electrophoresis through 2% agarose gel under UV light. Positive controls were set up using the recombinant plasmids containing the corresponding target genes (see supporting information for the construction of recombinant plasmids), while sterile water was used as the negative control. To prevent false negative results, PCR primers AllBac targeting the total intestinal flora was used to confirm PCR amplification of each fecal sample^33^. In addition, to avoid possible false negative results caused by potential PCR inhibitors, fecal samples that gave negative results were further checked after diluted five folds. To determine the detection limits of the PCR assays, corresponding recombinant plasmid DNAs with known concentrations were serially diluted and the lowest concentration of plasmid DNA detected in all triplicate tests was considered as the PCR detection limit (Table S12).

### Fecal abundances of DNA markers and quantitative PCR (qPCR)

The abundance of each DNA marker in feces was measured by the corresponding SYBR Green-based qPCR assay. The human (n = 19) and pig (n = 24) fecal samples which were positive for all the target markers (the 5 human-associated or the 4 pig-associated markers) were used for the measurements.

The quantitative real-time PCR (qPCR) assays were performed using a Rotor-Gene 6000 with the Rotor-Gene 6000 Series Software 1.7 (QIAGEN, the Netherlands). Each reaction was run in triplicate with a final volume of 20 μl containing 10 μl SYBR Premix^EX^ Taq Super Mix (TaKaRa Japan), plus 0.5 μl of each primer (250 nM), 5 μl of ddH_2_O and 4 μl of template DNA. The primers of the DNA markers for qPCR were listed in Table 4, which were the same as those for conventional-PCR. The corresponding qPCR programs are listed in Table S13. The corresponding standard curve of each qPCR assay was generated using 10-fold serial dilutions (10^8^–10^1^) of the recombinant plasmids carrying the target markers. Ct value of a test sample was used to calculate the abundance of each DNA marker. DNA melting curve analysis was performed to check for non-specific amplification in each assay. Amplified products were also visualized by electrophoresis through 2% agarose gel for further confirmation. All the qPCR assays had the amplification efficiency between 85% to 105% and R^2^ more than 0.99. The detection limits of the qPCR assays were 10 copies per reaction, which corresponds to the lowest concentration of the standard curve.

### Data analysis

The sensitivity and specificity of each DNA marker were calculated based on the results of the conventional PCR assays, as described by Gawler, *et al*.^34^ and the following equations were used: sensitivity = a/(a + c), where “a” is the number of positive samples and “c” is the number of negative samples when target fecal samples were used; specificity = b/(b + d), where “d” is the number of negative samples and “b” is the number of positive samples when non-target fecal samples were used.

The decay rate (k) of a DNA marker was estimated as the slope of the regression line following the first-order decay model ln(C_t_/C_0_) = −kt^35^, where “C_0_” is the initial concentration of the target DNA marker (copies/L); “C_t_” is the marker concentration at time “t” (copies/L); “t” is the incubation time in day (d); and “k” is the decay rate (d^−1^). The duration of incubation time (d) needed to obtain a 90% reduction from the initial concentration of the DNA marker was calculated as T_90_ = −ln(0.1)/k.

One-way ANOVA was performed to determine the statistical difference between different marker concentrations in fecal samples, and different marker decay rates under a certain condition, as well as decay rates of each marker at different temperatures (8 °C, 20 °C, and 30 °C). Two-way ANOVA was used to identify whether influent/effluent or seasons had significant effect on marker concentrations in SWTP samples^36^. Student’s *t* test was utilized to determine the statistical differences between T_90_ values of each marker in summer and winter, as well between 20 °C in dark and in light. All statistical analyses were performed by SPSS 15 software (SPSS Inc., USA), and a *p* value of two sides less than 0.05 was considered statistically significant.

## Additional Information

**How to cite this article**: He, X. *et al*. Evaluation of five microbial and four mitochondrial DNA markers for tracking human and pig fecal pollution in freshwater. *Sci. Rep.* **6**, 35311; doi: 10.1038/srep35311 (2016).

## Supplementary Material

Supplementary Information

## Figures and Tables

**Table 1:** Specificity and sensitivity of the human- and pig-associated DNA markers.

Sample source	No. of samples	No. of samples positive									
		AllBac	Human-associated markers					Pig-associated markers			
			BacH	HF183	B.adolescentis	H-ND6	H-ND5	Pig-2-Bac	L.amylovorus	P-CytB	P-ND5
Human	25	25	23	21	21	25	25	0	4	13	5
Pig	25	25	6	2	20	1	1	25	24	25	25
Bovine	8	8	1	0	2	0	0	0	0	4	2
Goat	10	10	5	5	5	0	0	0	0	6	3
Dog	8	8	6	2	2	0	0	0	0	8	6
Chicken	18	18	10	2	4	0	1	0	9	0	0
Duck	18	18	6	1	8	0	0	0	6	1	0
Goose	12	12	3	0	5	0	0	0	5	1	0
Cormorant	8	8	4	0	0	0	0	0	0	1	1
Total No. of samples	132										
No. of false positive samples			41	12	46	1	2	0	24	34	17
Specificity			61.7%	88.8%	57.0%	99.1%	98.1%	100%	74.8%	68.2%	84.1%
Sensitivity			92.0%	84.0%	84.0%	100%	100%	100%	96%	100%	100%

**Table 2:** Concentrations of the human-associated DNA markers in the SWTP samples.

Sampling sites	BacH		HF183		B.adolescentis		H-ND6		H-ND5	
	Winter	Summer	Winter	Summer	Winter	Summer	Winter	Summer	Winter	Summer
SWTP influent	8.52 ± 0.12	9.05 ± 0.10	8.06 ± 0.04	8.30 ± 0.01	8.49 ± 0.02	8.85 ± 0.20	7.35 ± 0.10	7.80 ± 0.04	6.07 ± 0.05	6.12 ± 0.06
SWTP effluent	6.58 ± 0.08	5.29 ± 0.40	6.35 ± 0.07	5.00 ± 0.06	6.81 ± 0.10	6.27 ± 0.06	5.62 ± 0.01	5.70 ± 0.03	4.01 ± 0.13	4.14 ± 0.13

Data are shown as log_10_ copy number per liter (mean ± standard deviation).

**Table 3:** T_90_ values of the DNA markers under different conditions

Host	Marker	Laboratory experiments				Field experiments	
		8 °C	20 °C	30 °C	Light (20 °C)	Winter	Summer
Human	BacH	22.71 ± 5.80	4.95 ± 0.13	3.38 ± 0.10	2.91 ± 0.01	3.56 ± 0.42	0.87 ± 0.12
	HF183	21.12 ± 6.36	4.44 ± 0.26	3.47 ± 0.30	2.45 ± 0.04	3.47 ± 0.32	0.76 ± 0.08
	B.adolescentis	16.29 ± 0.90	9.56 ± 0.79	6.34 ± 0.42	2.87 ± 0.10	4.90 ± 0.51	1.43 ± 0.21
	H-ND6	10.64 ± 0.69	5.78 ± 0.47	4.80 ± 0.30	1.71 ± 0.06	3.64 ± 0.38	0.87 ± 0.21
	H-ND5	11.51 ± 2.16	6.65 ± 0.79	5.32 ± 0.68	2.19 ± 0.07	3.64 ± 0.44	0.78 ± 0.15
Pig	Pig-2-Bac	9.76 ± 0.81	5.11 ± 0.30	3.49 ± 0.03	2.16 ± 0.04	2.51 ± 0.24	0.90 ± 0.06
	L.amylovorus	12.11 ± 2.15	10.93 ± 1.09	9.85 ± 2.65	2.67 ± 0.10	4.22 ± 0.50	1.05 ± 0.09
	P-CytB	7.15 ± 0.67	4.33 ± 0.26	3.03 ± 0.11	2.41 ± 0.02	3.20 ± 0.22	0.84 ± 0.08
	P-ND5	6.34 ± 0.62	4.40 ± 0.18	3.25 ± 0.04	2.42 ± 0.05	3.17 ± 0.10	0.80 ± 0.06

Data are shown as mean ± standard deviation.

**Table 4:** Information of the human- and pig-associated DNA markers.

Host	Marker name	Origin	Amplicon size (bp)	Primer (5′-3′)	Reference
Human	BacH	*Bacteroides-Prevotella*	93	Forward: CTTGGCCAGCCTTCTGAAAG Reverse: CCCCATCGTCTACCGAAAATAC	24
	HF183	*Bacteroides-Prevotella*	541	Forward: ATCATGAGTTCACATGTCCG Reverse: CAATCGGAGTTCTTCGTG	^8^
	B.adolescentis	*Bifidobacterium adolescentis*	324	Forward: GGGTGGTAATGCCGGATG Reverse: GGTGCTTATTCGAAAGGTACACTCA	^29^
	H-ND6	Mitochondrial DNA NADH 6 gene	97	Forward: GTTTACCACAACCACCACCC Reverse: GGTTGAGGTCTTGGTGAGTG	This study
	H-ND5	Mitochondrial DNA NADH 5 gene	195	Forward: CAGCAGCCATTCAAGCAATGC Reverse: GGTGGAGACCTAATTGGGCTGATTAG	27
Pig	Pig-2-Bac	*Bacteroidales*	116	Forward: GCATGAATTTAGCTTGCTAAATTTGAT Reverse: ACCTCATACGGTATTAATCCGC	25
	L.amylovorus	*Lactobacillus amylovorus*	152	Forward: TTCTGCCTTTTTGGGATCAA Reverse: CCTTGTTTATTCAAGTGGGTGA	37
	P-CytB	Mitochondrial DNA Cyt b gene	116	Forward: CGACAAAGCAACCCTCACACGATT Reverse: TAGGGTTGTTGGATCCGGTTTCGT	16
	P-ND5	Mitochondrial DNA NADH 5 gene	196	Forward: ACAGCTGCACTACAAGCAATGC Reverse: GGATGTAGTCCGAATTGAGCTGATTAT	27
