# Supplementary material for: Evaluation of five microbial and four mitochondrial DNA markers for tracking human and pig fecal pollution in freshwater
Source: Sci Rep. 2016 Oct 13;6:35311. doi: 10.1038/srep35311 (PMC5062121; doi:10.1038/srep35311)
Supplement: Supplementary Information [file srep35311-s1.doc]

**Supporting Information for**

**“Evaluation of five microbial and four mitochondrial DNA markers for tracking human and pig fecal pollution in freshwater”**

**Authors:**

Xiwei He^a^, Peng Liu^a^, Guolu Zheng^b^, Huimei Chen^c*^, Wei Shi^a^, Yibin Cui^a^, Hongqiang Ren^a^, Xu-Xiang Zhang^a*^

**Affiliations of authors:**

1. State Key Laboratory of Pollution Control and Resource Reuse, School of the Environment, Nanjing University, China.
2. College of Agriculture and Environmental Sciences, Lincoln University in Missouri, USA

c. Jiangsu Key Laboratory of Molecular Medicine, School of Medicine, Nanjing University, China

***Corresponding authors**

**Submitted to *Scientific Reports***

**Contents**

**S1 Method**

**Method S1** Construction of recombinant plasmids

**S2 Tables and Figures**

**Figure S1** Alignments of the pig mtDNA markers’ PCR primers with mtDNA sequences of other vertebrates.

**Table S1** *p* values based on one-way ANOVA to determine significant difference between the concentrations of markers in fecal samples.

**Table S2** Decay rates of the human- and pig-associated DNA markers. .

**Table S3** *p* values based on one-way ANOVA to determine significant differences between decay rates of the DNA markers under 8^o^C and dark treatment.

**Table S4** *p* values based on one-way ANOVA to determine significant differences between decay rates of the DNA markers under 20^o^C and dark treatment.

**Table S5** *p* values based on one-way ANOVA to determine significant differences between decay rates of the DNA markers under 30^o^C and dark treatment.

**Table S6** *p* values based on one-way ANOVA to determine significant differences between decay rates of the DNA markers under 20^o^C and light treatment.

**Table S7** *p* values based on one-way ANOVA to determine significant differences between decay rates of the DNA markers in the winter field experiment.

**Table S8** *p* values based on one-way ANOVA to determine significant differences between decay rates of the DNA markers in the summer field experiment.

**Table S9** Concentrations of the human-associated DNA markers in Taige River water samples.

**Table S10** Basic daytime weather and river water qualities (Taige River) at each sampling time.

**Table S11** The thermocycle conditions for the conventional-PCR amplifications.

**Table S12** Detection limits of the conventional-PCR assays for the markers.

**Table S13** The thermocycle conditions for the qPCR amplifications.

**S1 Method**

**Construction of recombinant plasmids**

The human-associated DNA markers (BacH, HF183, B.adolescentis, H-ND6, and H-ND5) and pig-associated DNA markers (Pig-2-Bac, L.amylovorus, P-CytB, and P-ND5) were isolated from human and pig feces, respectively. The PCR product of each DNA marker was purified using the Takara MiniBEST DNA Fragment Purification Kit (TaKaRa, Japan) and cloned into the pMD19-T Vector (TaKaRa, Japan), transformed into *E. coli* Trans1-T1 Chemically Comptent Cell (TransGen Biotech, China), and plated on LB agar plates containing ampicillin, IPTG, and X-gal as recommended by the manufacturer. Recombinant plasmids with corresponding inserts were purified using Takara MiniBEST Plasmid Purification Kit (Takara, Japan). DNA sequencing was carried out at the Beijing Genomic Institute (Beijing, China).

**S2 Tables and Figures**


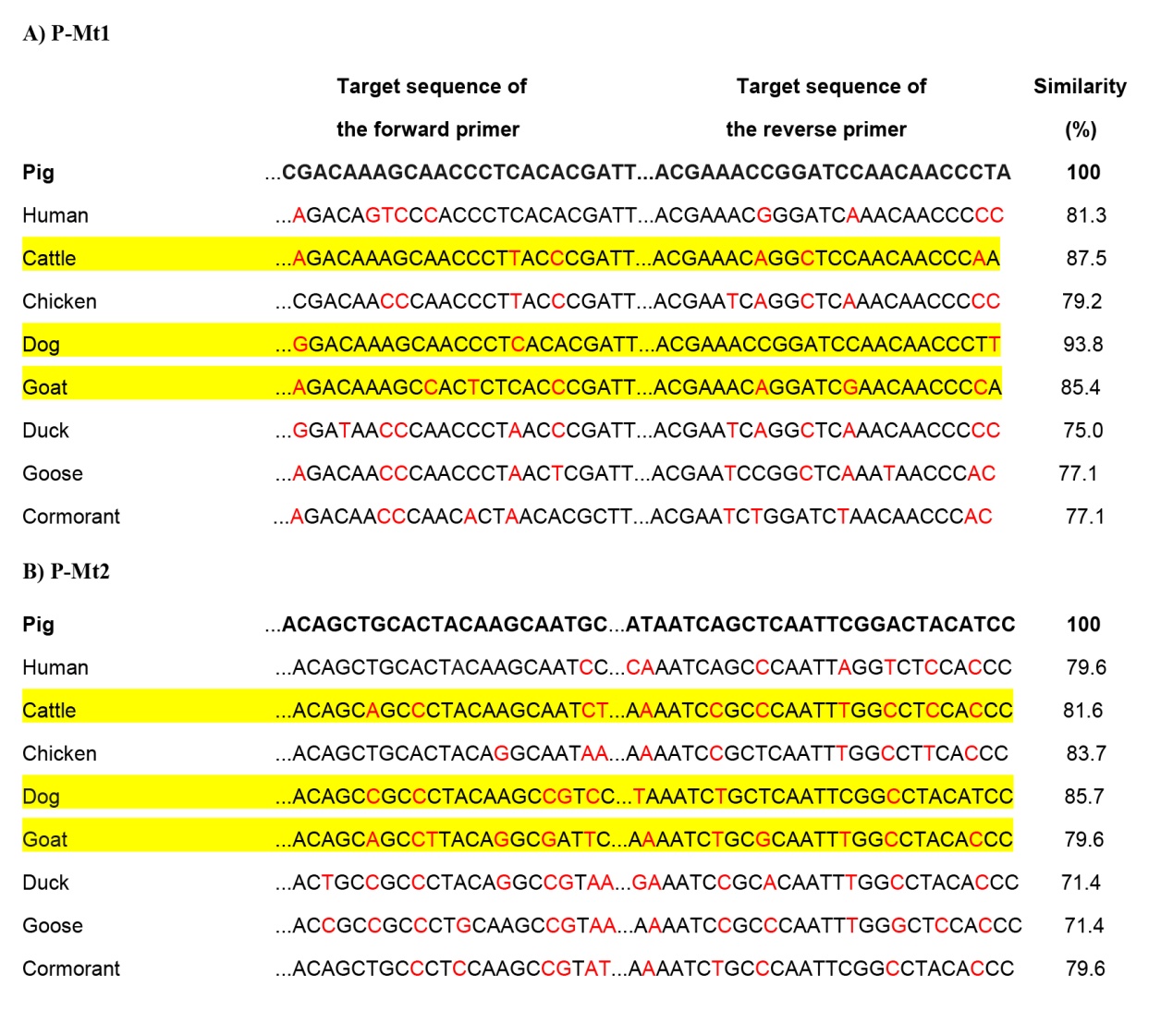


**Figure S1** Alignments of the PCR primers for marker P-CytB (A) and marker P-ND5All (B). The mtDNA sequences are from the GenBank: human, gi|308206849|; bovine, gi|294679591|; chicken, gi|700587922|; dog, gi|393007548|; goat, gi|756419391|; duck, gi|675023238|; goose, gi|817597733|; and cormorant, gi|827346607|.

**Table S1** *p* values based on one-way ANOVA to determine significant difference between the concentrations of markers in fecal samples.

| Human markers | BacH | HF183 | *B. adolescentis* | H-ND6 | H-ND5 |
| --- | --- | --- | --- | --- | --- |
| BacH | 1.000 | **<0.001** | **0.001** | **0.003** | **<0.001** |
| HF183 |  | 1.000 | 0.606 | 0.396 | **0.044** |
| *B. adolescentis* |  |  | 1.000 | 0.739 | **0.012** |
| H-ND6 |  |  |  | 1.000 | **0.005** |
| H-ND5 |  |  |  |  | 1.000 |
| Pig markers | Pig-2-Bac | L.amylovorus | P-CytB | P-ND5 |  |
| Pig-2-Bac | 1.000 | **<0.001** | **<0.001** | **<0.001** |  |
| L.amylovorus |  | 1.000 | 0.304 | **0.012** |  |
| P-CytB |  |  | 1.000 | **0.001** |  |
| P-ND5 |  |  |  | 1.000 |  |

**Table S2** Decay rates of the human- and pig-associated DNA markers.

| **Host** | **Marker** | **Laboratory experiments** | | | |  | **Field experiments** | |
| --- | --- | --- | --- | --- | --- | --- | --- | --- |
|  |  | **8℃** | **20℃** | **30℃** | **Light (20℃)** |  | **Winter** | **Summer** |
| Human | BacH | 0.11 ±0.03 | 0.47 ±0.01 | 0.68 ±0.02 | 0.79 ±0.01 |  | 0.65 ±0.07 | 2.70 ±0.40 |
|  | HF183 | 0.12 ±0.03 | 0.52 ±0.03 | 0.67 ±0.06 | 0.94 ±0.02 |  | 0.67 ±0.06 | 3.07 ±0.34 |
|  | B.adolescentis | 0.14 ±0.01 | 0.24 ±0.02 | 0.36 ±0.02 | 0.80 ±0.03 |  | 0.47 ±0.05 | 1.64 ±0.25 |
|  | H-ND6 | 0.22 ±0.01 | 0.40 ±0.03 | 0.48 ±0.03 | 1.34 ±0.04 |  | 0.64 ±0.06 | 2.75 ±0.59 |
|  | H-ND5 | 0.21 ±0.04 | 0.35 ±0.04 | 0.44 ±0.05 | 1.05 ±0.03 |  | 0.64 ±0.08 | 3.02 ±0.60 |
|  |  |  |  |  |  |  |  |  |
| Pig | Pig-2-Bac | 0.24 ±0.02 | 0.45 ±0.03 | 0.66 ±0.01 | 1.07 ±0.02 |  | 0.92 ±0.08 | 2.56 ±0.19 |
|  | L.amylovorus | 0.19 ±0.04 | 0.21 ±0.02 | 0.25 ±0.08 | 0.86 ±0.03 |  | 0.55 ±0.06 | 2.21 ±0.19 |
|  | P-CytB | 0.32 ±0.03 | 0.53 ±0.03 | 0.76 ±0.03 | 0.96 ±0.01 |  | 0.72 ±0.05 | 2.76 ±0.28 |
|  | P-ND5 | 0.37 ±0.03 | 0.52 ±0.02 | 0.71 ±0.01 | 0.95 ±0.02 |  | 0.73 ±0.02 | 2.87 ±0.21 |

Data are shown as mean ± standard deviation

**Table S3** *p* values based on one-way ANOVA to determine significant differences between decay rates of the DNA markers under 8^o^C and dark treatment.

| Human markers | HF183 | B.adolescentis | H-ND6 | H-ND5 |
| --- | --- | --- | --- | --- |
| BacH | 0.702 | 0.139 | **0.001** | **0.001** |
| HF183 |  | 0.253 | **0.001** | **0.002** |
| B.adolescentis |  |  | **0.007** | **0.016** |
| H-ND6 |  |  |  | 0.597 |
| Pig markers | L.amylovorus | P-CytB | P-ND5 |  |
| Pig-2-Bac | 0.125 | **0.008** | **0.001** |  |
| L.amylovorus |  | **0.001** | **0.000** |  |
| P-CytB |  |  | 0.130 |  |

**Table S4** *p* values based on one-way ANOVA to determine significant differences between decay rates of the DNA markers under 20^o^C and dark treatment.

| Human markers | HF183 | B.adolescentis | H-ND6 | H-ND5 |
| --- | --- | --- | --- | --- |
| BacH | 0.052 | **0.000** | **0.023** | **0.001** |
| HF183 |  | **0.000** | **0.001** | **0.000** |
| B.adolescentis |  |  | **0.000** | **0.001** |
| H-ND6 |  |  |  | 0.064 |
| Pig markers | L.amylovorus | P-CytB | P-ND5 |  |
| Pig-2-Bac | **0.000** | **0.004** | **0.008** |  |
| L.amylovorus |  | **0.000** | **0.000** |  |
| P-CytB |  |  | 0.675 |  |

**Table S5** *p* values based on one-way ANOVA to determine significant differences between decay rates of the DNA markers under 30^o^C and dark treatment.

| Human markers | HF183 | B.adolescentis | H-ND6 | H-ND5 |
| --- | --- | --- | --- | --- |
| BacH | 0.641 | **0.000** | **0.000** | **0.000** |
| HF183 |  | **0.000** | **0.000** | **0.000** |
| B.adolescentis |  |  | **0.006** | **0.053** |
| H-ND6 |  |  |  | 0.219 |
| Pig markers | L.amylovorus | P-CytB | P-ND5 |  |
| Pig-2-Bac | **0.000** | **0.018** | 0.188 |  |
| L.amylovorus |  | **0.000** | **0.000** |  |
| P-CytB |  |  | 0.168 |  |

**Table S6** *p* values based on one-way ANOVA to determine significant differences between decay rates of the DNA markers under 20^o^C and light treatment.

| Human markers | HF183 | B.adolescentis | H-ND6 | H-ND5 |
| --- | --- | --- | --- | --- |
| BacH | **0.000** | 0.582 | **0.000** | **0.000** |
| HF183 |  | **0.000** | **0.000** | **0.001** |
| B.adolescentis |  |  | **0.000** | **0.000** |
| H-ND6 |  |  |  | **0.000** |
| Pig markers | L.amylovorus | P-CytB | P-ND5 |  |
| Pig-2-Bac | **0.000** | **0.000** | **0.000** |  |
| L.amylovorus |  | **0.001** | **0.001** |  |
| P-CytB |  |  | 0.859 |  |

**Table S7** *p* values based on one-way ANOVA to determine significant differences between decay rates of the DNA markers in the winter field experiment.

| Human markers | HF183 | B.adolescentis | H-ND6 | H-ND5 |
| --- | --- | --- | --- | --- |
| BacH | 0.798 | **0.007** | 0.770 | 0.788 |
| HF183 |  | **0.005** | 0.585 | 0.601 |
| B.adolescentis |  |  | **0.012** | **0.011** |
| H-ND6 |  |  |  | 0.980 |
| Pig markers | L.amylovorus | P-CytB | P-ND5 |  |
| Pig-2-Bac | **0.000** | **0.003** | **0.004** |  |
| L.amylovorus |  | **0.008** | **0.007** |  |
| P-CytB |  |  | 0.914 |  |

**Table S8** *p* values based on one-way ANOVA to determine significant differences between decay rates of the DNA markers in the summer field experiment.

| Human markers | HF183 | B.adolescentis | H-ND6 | H-ND5 |
| --- | --- | --- | --- | --- |
| BacH | 0.348 | **0.017** | 0.887 | 0.416 |
| HF183 |  | **0.003** | 0.421 | 0.895 |
| B.adolescentis |  |  | **0.014** | **0.004** |
| H-ND6 |  |  |  | 0.498 |
| Pig markers | L.amylovorus | P-CytB | P-ND5 |  |
| Pig-2-Bac | 0.089 | 0.296 | 0.122 |  |
| L.amylovorus |  | **0.016** | **0.006** |  |
| P-CytB |  |  | 0.556 |  |

**Table S9 Concentrations of the human-associated DNA markers in Taige River water samples.**

| Sampling sites | BacH | | HF183 | | | *B. adolescentis* | | | H-ND6 | | H-ND5 | |
| --- | --- | --- | --- | --- | --- | --- | --- | --- | --- | --- | --- | --- |
|  | Winter | Summer | | Winter | Summer | | Winter | Summer | Winter | Summer | Winter | Summer |
| TG1 | 5.11±0.11 | 4.23±0.17 | | 4.62±0.15 | 4.30±0.20 | | 5.09±0.18 | ND | 4.81±0.17 | ND | 3.54±0.17 | ND |
| TG2 | 4.84±0.30 | ND | | 4.08±0.14 | 3.90±0.06 | | 5.96±0.18 | 3.80±0.12 | 4.87±0.08 | ND | 4.12±0.04 | ND |
| TG3 | 5.68±0.43 | 3.63±0.36 | | 4.89±0.02 | 3.61±0.04 | | 5.95±0.16 | 5.03±0.15 | 4.88±0.01 | 4.49±0.48 | 4.22±0.08 | 3.63±0.43 |
| TG4 | 5.27±0.09 | 3.57±0.41 | | 4.84±0.29 | 3.18±0.28 | | 5.73±0.16 | 4.35±0.39 | 5.36±0.16 | 4.73±0.30 | 3.84±0.21 | 3.62±0.06 |
| TG5 | 4.76±0.11 | 4.72±0.33 | | 4.69±0.05 | 4.04±0.30 | | 5.13±0.16 | ND | 5.15±0.01 | ND | 3.37±0.27 | ND |
| TG6 | 5.01±0.13 | ND | | 4.80±0.04 | 4.40±0.36 | | 5.30±0.25 | ND | 4.77±0.08 | ND | 3.92±0.10 | ND |
| TG7 | 5.70±0.33 | 3.33±0.49 | | 4.86±0.06 | ND | | 4.69±0.01 | 4.26±0.10 | 4.62±0.20 | 4.42±0.14 | 3.93±0.33 | 3.77±0.26 |
| TG8 | 4.46±0.11 | 3.22±0.34 | | 4.10±0.03 | ND | | 4.31±0.04 | 4.08±0.20 | 5.36±0.01 | ND | 3.75±0.19 | ND |
| TG19 | 4.23±0.21 | 4.07±0.55 | | 4.05±0.01 | 3.63±0.36 | | 5.09±0.11 | 4.00±0.35 | 4.69±0.34 | 3.97±0.23 | 3.53±0.08 | 3.83±1.28 |
| TG10 | 6.65±0.10 | ND | | 5.35±0.09 | ND | | 5.50±0.69 | ND | 4.94±0.03 | 4.36±0.08 | 4.25±0.19 | 3.54±0.04 |
| TG11 | 4.63±0.14 | ND | | 4.07±0.55 | ND | | 4.66±0.22 | ND | 3.61±0.07 | 3.82±0.52 | ND | ND |
| TG12 | 4.63±0.09 | ND | | 4.09±0.10 | ND | | 5.11±0.05 | ND | 4.85±0.13 | ND | 3.20±0.11 | ND |
| Detection rate in river water | 100% | 58.3% | | 100% | 58.3% | | 100% | 50% | 100% | 50% | 91.7% | 41.7% |

Data are shown as log_10_ copy number per liter (mean ± standard deviation)

ND, not detectable

**Table S 10** Basic daytime weather and river water qualities (Taige River) at each sampling time.

| Time | Daytime Weather | Temperature (°C) | pH | DO (mg/L) | Turbidity (NTU) | Salinity (ng/L) |
| --- | --- | --- | --- | --- | --- | --- |
| **Summer** |  |  |  |  |  |  |
| 9.1 0:00 | Sunny | 27.95 | 7.17 | 6.05 | 2.1 | 0.14 |
| 9.1 6:00 |  | 27.25 | 6.98 | 12.5 | 2.7 | 0.14 |
| 9.1 12:00 |  | 29.17 | 8.06 | 9.59 | 11.1 | 0.13 |
| 9.1 18:00 |  | 28.34 | 7.29 | 7.52 | 2.6 | 0.14 |
| 9.1 24:00 |  | 27.36 | 7.09 | 5.08 | 2.0 | 0.14 |
| 9.2 24:00 | Sunny | 27.90 | 7.11 | 6.03 | 2.2 | 0.14 |
| **Winter** |  |  |  |  |  |  |
| 11.21 0:00 | Sunny | 12.89 | 6.54 | 0.79 | 12.0 | 0.20 |
| 11.22 0:00 | Cloudy | 13.16 | 6.90 | 1.57 | 2.2 | 0.19 |
| 11.23 0:00 | Rainy | 14.25 | 6.96 | 2.03 | 2.2 | 0.20 |
| 11.24 0:00 | Sunny | 12.69 | 7.02 | 1.64 | 4.3 | 0.19 |
| 11.25 0:00 | Cloudy | 12.34 | 6.92 | 1.66 | 4.2 | 0.20 |
| 11.26 0:00 | Sunny | 12.41 | 6.96 | 1.56 | 3.6 | 0.20 |
| 11.27 0:00 | Sunny | 11.38 | 7.03 | 1.86 | 5.0 | 0.20 |

**Table S11 The thermocycle conditions for the conventional-PCR amplifications.**

| Marker | Initial denaturation | Denaturation | Anneal | Extension | Final extention |
| --- | --- | --- | --- | --- | --- |
|  |  | 40cycles | | |  |
| HF183 | 95°C, 15min | 94°C, 30sec | 59°C, 60sec | 72°C, 60sec | 72°C, 7min |
| BacH | 95°C, 3min | 95°C, 15sec | 61°C, 45sec | 72°C, 45sec | 72°C, 7min |
| B.adolescentis | 95°C, 10min | 95°C, 15sec | 60°C, 60sec | 72°C, 30sec | 72°C, 7min |
| H-ND5 | 95°C, 2min | 94°C, 10sec | 60°C, 12sec | 72°C, 10sec | 72°C, 7min |
| H-ND6 | 95°C, 30sec | 95°C, 5sec | 59°C, 30sec | 72°C, 30sec | 72°C, 7min |
| Pig-2-Bac | 95°C, 30sec | 95°C, 5sec | 60°C, 30sec | 72°C, 30sec | 72°C, 7min |
| L.amylovorus | 95°C, 3min | 95°C, 15sec | 59°C, 45sec | 72°C, 30sec | 72°C, 7min |
| P-ND5 | 95°C, 2min | 94°C, 10sec | 60°C, 12sec | 72°C, 10sec | 72°C, 7min |
| P-CytB | 95°C, 10min | 95°C, 10sec | 60°C, 15sec | 72°C, 20sec | 72°C, 7min |

**Table S12** Detection limits of the conventional-PCR assays for the markers.

| Target host | Marker | Detection limit (copies per reaction) |
| --- | --- | --- |
| Human | BacH | 30 |
|  | HF183 | 23 |
|  | B.adolescentis | 21 |
|  | H-ND6 | 25 |
|  | H-ND5 | 16 |
| Pig | Pig-2-Bac | 27 |
|  | L.amylovorus | 20 |
|  | P-CytB | 34 |
|  | P-ND5 | 29 |

**Table S13** The thermocycle conditions for the qPCR amplifications.

| Marker | Initial hold | Initial denaturation | Denaturation | Anneal | Extension |
| --- | --- | --- | --- | --- | --- |
|  |  |  | 40cycles | | |
| HF183 | 50°C,2min | 95°C, 15min | 94°C, 30sec | 59°C, 60sec | 72°C, 60sec |
| BacH |  | 95°C, 3min | 95°C, 15sec | 61°C, 45sec | 72°C, 45sec |
| B.adolescentis |  | 95°C, 10min | 95°C, 15sec | 60°C, 60sec |  |
| H-ND5 |  | 95°C, 2min | 94°C, 10sec | 60°C, 12sec | 72°C, 10sec |
| H-ND6 |  | 95°C, 30sec | 95°C, 5sec | 59°C, 30sec |  |
| Pig-2-Bac |  | 95°C, 30sec | 95°C, 5sec | 60°C, 30sec |  |
| L.amylovorus |  | 95°C, 3min | 95°C, 15sec | 59°C, 45sec |  |
| P-ND5 |  | 95°C, 2min | 94°C, 10sec | 60°C, 12sec | 72°C, 10sec |
| P-CytB |  | 95°C, 10min | 95°C, 10sec | 60°C, 15sec | 72°C, 20sec |
